# Tribe at a Crossroads: The Navajo Nation Purchases a Coal Mine

**DOI:** 10.1289/ehp.122-A104

**Published:** 2014-04-01

**Authors:** Rebecca Fairfax Clay

**Affiliations:** **Rebecca Fairfax Clay** is a freelance writer and has contributed to *EHP* since 1993. She lives in Santa Fe, NM.

**Figure:**
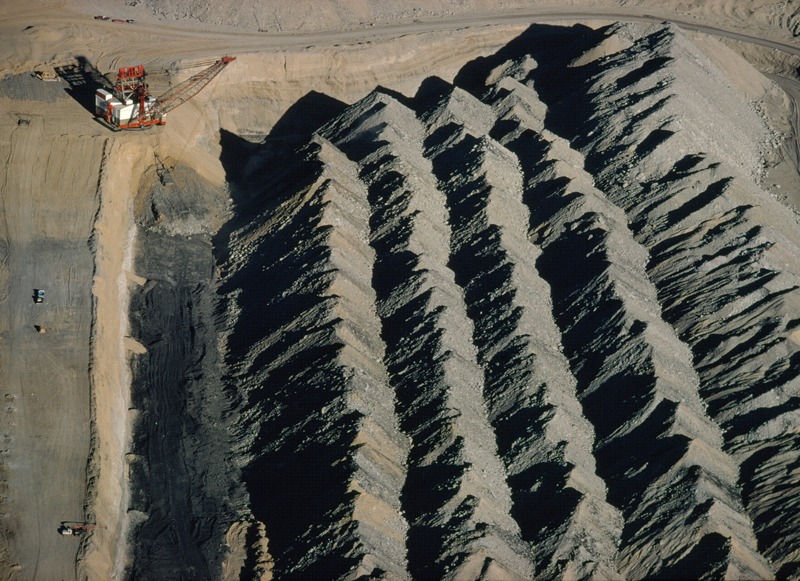
Aerial view of Navajo Mine, bought by the Navajo Nation in December 2013. © Bruce Dale/National Geographic Creative

Situated where the four corners of Arizona, Utah, New Mexico, and Colorado meet, the 27,000-square-mile Navajo Nation boasts some of the most abundant energy resources on tribal lands in the United States, including fossil fuels and the potential for using wind and sun. In December 2013 the tribe shifted for the first time from leasing much of its coal-rich land to outside mining companies, to owning and operating one of its coal mines itself.^1^ But the $85-million purchase has caused deep concern among critics who fear it saddles the tribe with the twin burdens of a polluted past and an unsustainable future.

The Navajo Nation bought Navajo Mine from Australian energy giant BHP Billiton, which reportedly is selling off smaller assets worldwide.^2^ The mine’s sole customer is the nearby Four Corners Power Plant (FCPP)—one of the largest coal-fired generating stations in the country—which, likewise, buys all its coal from Navajo Mine.^3^

If the 50-year-old mine had been shut down, as feared by tribal leaders, hundreds of jobs and millions in tax and royalty revenue for the Navajo Nation would have been at risk, according to Steve Gundersen, board chairman of the Navajo Transitional Energy Company, LLC, a company created by the tribe to help acquire and manage the mine. In addition, if the mine had been closed, the plant would have been expected to close as well.^4^

“The most immediate purpose for buying the mine was to preserve the stability of the Navajo Nation’s economy,” Gundersen says. “If the mine and power plant were removed from the Navajo economy, the results, within a year, would have been devastating. We needed to preserve the business and income.”

The purchase potentially offers another benefit, as LoRenzo Bates, chairman of the Navajo Nation Council’s Budget and Finance Committee, told *Indian Country Today*. “Rather than sitting on the sidelines, we now have a say in the energy industry in terms of how that reserve of coal is being used,” Bates said. “This … goes beyond the coal industry and allows us a voice in alternative forms of energy. Coal can have other uses, and this makes us a player in the industry.”^5^

Environmental groups acknowledge the importance of Navajo coal to the rapid growth of the U.S. Southwest, and the thousands of jobs it has provided in the remote and impoverished Four Corners area, where most of the region’s coal mines are located. But they are concerned about the environmental impacts of more than a half-century of coal operations.

“Coal has really been a building block for this part of the country,” says Mike Eisenfeld, New Mexico energy coordinator of the San Juan Citizens Alliance, an environmental group working in the region. “The Navajo Nation has been highly dependent on fossil fuels and fossil fuel electricity export, especially when you realize that a large portion of their budget is associated with coal. But, we ask, at what expense?”

## Jobs and Revenue versus the Environment

The tribe completed the mine purchase through seller financing, with BHP Billiton providing a loan for the purchase price to Navajo Transitional Energy Company. Proceeds from the revenue generated from the mine, beyond what the Navajo Nation would normally have received, will be applied toward the purchase price.^6^ Under the terms of the deal, BHP Billiton will continue to manage the mine through 2016, when the tribe—or a separate company—is expected to assume management, according to BHP Billiton New Mexico Coal president Pat Risner.

The sale would help “secure the significant flow of benefits to the community,” says Risner. “Navajo Mine is a viable business, and the Navajo Nation is the ‘natural owner,’ given it is a Navajo Nation resource on Navajo Nation land with primarily Navajo tribal members employed.”

But the purchase of Navajo Mine has pitted those hoping to preserve employment and income against those who argue the Navajo Nation should quit the coal business entirely and dev-elop small- and utility-scale sustainable energy projects instead. The mine’s purchase agreement includes a mandate to invest 10% of its net profits in renewable technologies such as solar and wind.^6^ Critics fear, however, that falling prices and demand for coal, both locally and globally, will reduce the mine’s future profits and therefore investments in renewables.^7^

“Putting all of their cards into fossil fuels … limits [the Navajo Nation’s] ability to develop renewable energies such as solar and wind,” Eisenfeld says. “And as coal loses its appeal worldwide, the tribe may find itself with a stranded asset and lose more than they gain.”

They also point to a waiver signed by Navajo leaders reportedly absolving BHP Billiton of past, present, and future liabilities, including the cost of cleanup.^8^ The mutual release agreement, which was appended to the legislation authorizing the purchase after two hours of debate,^9^ has not been made public, according to sources for this story. This is another reason Navajo activists are suspicious of their political leaders; they say the purchase was carried out with too much secrecy.^10^

“If liabilities bankrupt the Navajo Nation, we won’t have funds to develop cleaner energy,” says Lori Goodman, treasurer of Diné CARE, a Navajo environmental group fighting the tribe’s ongoing involvement in coal. “There are millions of tons of coal ash sitting out there by the mine, and it’s going to cost a huge amount to clean it up. How can we afford that? Our leaders pushed this deal through way too fast. It was all about jobs. ‘Do you want the people to lose their jobs? Buy the mine.’ In our opinion, this is a huge disaster waiting to happen.”

Gundersen argues the waiver of liability is standard practice in corporate transactions. “At some point,” he says, “the reason a company is selling an asset is to end its obligations. So while this is a nice talking point for environmentalists, the truth is that closing down coal mines and plants can be quite devastating to local economics. That is what the Navajo Nation is trying to avoid.”

Furthermore, Risner says, “BHP Billiton will continue to be subject to certain federal laws, such as the Comprehensive Environmental Response, Compensation, and Liability Act [i.e., Superfund], into the future with respect to certain liabilities should they arise.” However, the full terms and conditions of the sale are subject to a nondisclosure agreement.^1^

Defenders of the purchase say the tribe had little choice about the waiver. Unemployment on the Navajo Nation is estimated as high as 64%,^11^ with most employed Navajo working in the mining, government, retail, and service sectors. In a 31 October 2013 press release, Navajo Nation president Ben Shelly said the tribe must remain “vigilant to protect our vested interests in Navajo energy, and keep our eyes on the future because the work we do today is for our grandchildren.”^12^ (Attempts to reach Shelly for comment were unsuccessful.)

In late 2012, as reports of an impending mine purchase became public, Shelly’s former spokesman, Erny Zah, described the deal as being “about Navajo Nation sovereignty. We’re talking about owning our assets,” he told the Farmington *Daily Times*. “It’s definitely a positive for Navajo to assert control and ownership of the natural resources that are on the Navajo Nation.”^13^

Environmental groups say that before buying the mine, Navajo leaders should have waited for the U.S. Department of Interior’s Office of Surface Mining Reclamation and Enforcement (OSMRE) to issue its environmental impact statement for continued operation of the mine and power plant, a draft of which was published as this article was going to press.^14^

“We feel there’s a lot of information that could come out in that statement that is public health based,” says Eisenfeld. “By waiting for the results, Navajo leaders would have understood better what they were getting into and what liabilities might lie ahead. Signing that waiver makes them far more vulnerable.”

But the draft environmental impact statement would appear to offer relatively little cause for worry, at least as far as public health goes. According to the OSMRE analysis, most potential impacts—including air pollution, noise, waste spills, and groundwater contamination—are predicted to be negligible or minor at worst. The only major long-term impacts estimated for either the mine or the plant are disruption of historical resources and the altered appearance of the landscape.^14^

## Fifty Years of Navajo Coal

Located in northwestern New Mexico, the 33,000-acre Navajo Mine and nearby FCPP have fueled hundreds of thousands of homes in Arizona, New Mexico, Texas, and (until recently) California since the 1960s.^15^ Several hundred people, most of them Navajo, work at Navajo Mine and FCPP. The mine and plant together contribute more than $100 million in taxes, fees, and royalties to the tribe’s annual budget.^16^

Although some Navajo residents recently connected to the power grid, many continue to use kerosene, wood, gasoline-powered generators and even chunks of coal to light and heat their homes.^17^ According to Eisenfeld, none of the electricity from FCPP is slated to be directly provided to the Navajo Nation.

Sarah Jane White, a member of Tiis Tsoh Sikaad Chapter of the Navajo Nation, says the industrial use of fossil fuels contradicts ancient tribal beliefs in the sanctity of nature. “The coal companies abuse Mother Earth, and we let them,” says White, whose father was a medicine man. “The Navajo people are upset about them dynamiting the earth and digging out the coal because, also, what do we get in return? We have so much poverty, and so many people have no electricity themselves. You see? We don’t need these dirty mines and power plants.”

In 2012 Navajo Mine sent 7.6 million tons of coal to FCPP,^18^ which was cited by the U.S. Environmental Protection Agency (EPA) as the country’s biggest emitter of nitrogen oxides (NO_x_), a greenhouse gas and major component of ground-level ozone.^19^ NO_x_ emissions from FCPP and other regional power plants have been blamed for the brown haze that often hovers over the Grand Canyon and other national parks in the area.^20^

Late in 2013 Arizona Public Service (APS) Company, which manages and owns a share of FCPP, shut down its three oldest boilers to meet EPA requirements for NO_x_ emissions.^16^ APS also plans to retrofit its remaining two units with cleaner technologies in the next few years.^16^ These changes—as well as Southern California Edison’s decision to sell its own share in the plant—are expected to improve regional air, soil, and water in the coming years.^21^

According to APS, the plant will downsize from 2,100 megawatts to 1,540 megawatts, hoping to trim NO_x_ emissions by 36%, mercury by 61%, particulate matter by 43%, carbon dioxide by 30%, and sulfur dioxide by 24%.^16^ These efficiencies will also reduce demand for Navajo Mine’s coal, thereby impacting employment at the mine; however, BHP Billiton has stated that the 100–200 mine positions lost “can be accomplished through retirement and attrition without layoffs.”^4^ No layoffs are expected at FCPP.^16^

## Other Facilities in the Region

The Four Corners region is home to additional coal mines and coal-fired power plants, some of which have been shuttered in recent years due to environmental and health concerns. Among those still in operation, San Juan Mine continues to be owned by BHP Billiton. This mine has sent coal to the nearby San Juan Generating Station since 1973, including nearly 5 million tons in 2012.^18^ The power plant has been cited by the EPA for emissions of NO_x_, sulfur dioxide, mercury, lead, chromium, and nickel that violate regional haze requirements. Its owners—a group of regional power utilities—plan to shut down two of the plant’s units and install NO_x_-reducing technology on the remaining two in the next few years.^22^

Kayenta Mine, which is owned by Peabody Energy Company, sent more than 7 million tons of coal to Navajo Generating Station in 2012.^18^ Both mine and plant have supplied energy to Arizona, Nevada, and California since the 1970s—between them, they employ about 900 workers, mostly Navajo. Navajo Generating Station was also cited by the EPA for excessive emissions of NO_x_ and other pollutants, and its owners are now planning steps to reduce emissions from the plant’s three boilers. In the meantime, the Navajo Nation Council extended the lease for operations at Navajo Generating Station through 2044, upping annual receipts to the tribe from about $600,000 to $42 million.^23^

Another coal mine in the Four Corners area closed in 2005 when the power plant it served, Nevada’s Mohave Generating Station, shut down rather than install updated pollution controls that would have cost more than $1 billion.^24^ The now-defunct Black Mesa Mine was the only mine in the United States to use a slurry process, which involves mixing pulverized coal with water and pumping it through a pipeline to the power plant.

Controversial from the start, still an-other coal-fired power plant at Four Corners never got off the ground. Navajo officials had hoped the Desert Rock Energy Facility, which would have been one of the largest generating stations in the country, would bring in $50 million in tribal revenue by providing power to heavily populated Las Vegas and Phoenix. Desert Rock was, in later stages of permitting, supposed to use “clean” technologies, including carbon sequestration, which captures and stores carbon dioxide. Amid concerns that the plant would still have emitted 12 million tons of carbon dioxide each year,^25^ the EPA revoked its air permit in 2009.^26^ That same year, San Juan County, where the plant is located, withdrew industrial bonds slated to fund construction of Desert Rock because of the lack of permits.^27^

“When our leaders asked us to pass a resolution accepting a power plant in Desert Rock, I said no,” White says. “We already have two power plants; that’s enough. It’s so smoky that sometimes we can’t even see. I said, ‘What kind of future do you want our children and grandchildren to have? Do you want them to sit in the smoke?’ They say they want to save the jobs for the young people, but many of them are going off to college. Why would they save these dirty mines and power plants for our kids? We don’t want them working in the mine.”

Complaints of breathing difficulties from Navajo residents near the mines and power plants are anecdotal—no research has been published on this association, although one study assessed the effects of indoor coal burning.^28^ “We need health studies,” says Dailan Long, a consultant for the New Mexico Medicaid Self-Directed Waiver Program and board member of Diné CARE. “The dollars that went to buying Navajo Mine could have gone back into the community for health needs. This purchase is very unfortunate. Nowhere else is anyone buying a mine—they’re selling them instead.”

## Developing Sustainable Energy

In October 2013 tribal leaders signed a new Navajo Nation Energy Policy, updating a version from 1980.^29^ The new policy was created to provide guidance to both Navajo and non-Navajo leaders for the development of fossil fuels and renewable energy sources.

Gundersen says that over the years Navajo leaders have been “inundated” with proposals for wind, solar, and other alternative energy projects. “Many were from individuals with no expertise and fell short on many fronts,” he says. “But what we see now is a changing dynamic—the Navajo will initiate projects themselves and consider only those that would optimize benefits to the tribe and its people.”

One solar project in its early stages is the proposed Paragon-Bisti Renewable Energy Ranch. The U.S. Department of Energy is funding feasibility studies for the mainly photovoltaic project, which could become one of the largest solar ranches in the world, covering more than 22,000 acres of Navajo land and producing more than 4,000 megawatts of power.^30^

But even solar energy can adversely affect the environment, Gundersen cautions. For a photovoltaic facility, he explains, “you have to cover thousands of acres with glass panels, spray chemicals on pristine land to suppress plant growth, and surround the whole thing with a big fence. As with coal, not every community wants that.”

Announced in 2009, the Boquillas Wind Project is one of several proposed large-scale wind operations on Navajo land. The facility would be the first renewable energy project majority-owned by a Native American tribe—the Navajo would own 51%—and could power thousands of homes around Phoenix and on the Navajo Nation. Building the wind farm is expected to employ up to 350 people, but just 10 employees would be needed to manage it when finished.^31^

Despite these pending projects, some Navajo remain skeptical of tribal leaders’ commitment to replacing coal with alternative energy projects. “Our tribe’s been giving lip service to renewables,” says Goodman. “We need a whole new crop of younger people to take over, because it’s not going to happen [with existing leadership]. If we’re one of the ten richest tribes in America, why are we also one of the poorest? If we were truly forward-looking people, they really would be acting in the best interests of the Navajo people.”
